# Correlation of Venous Clinical Severity Score With Dermatology Life Quality Index Among Patients With Chronic Venous Insufficiency: A Cross-Sectional Study

**DOI:** 10.7759/cureus.17654

**Published:** 2021-09-01

**Authors:** Divya Poulose, Kirti Deo, Jaya Madhurya Gogineni, Aditi Mahajan, Samruddhi Lote, Roshni Mishra, George Jacob, Mariet Zacharias, Abida Babu

**Affiliations:** 1 Department of Dermatology, Dr. D. Y. Patil Medical College, Hospital and Research Centre, Pune, IND; 2 Department of Oral and Maxillofacial Surgery, Dr. D. Y. Patil Dental College and Hospital, Pune, IND; 3 Department of Dermatology, Kempegowda Institute of Medical Sciences, Bangalore, IND; 4 Internal Medicine, University of South Florida Morsani College of Medicine, Tampa, USA

**Keywords:** chronic venous insufficiency, venous clinical severity score, dermatology life quality index, venous ulcers, varicose veins, lipodermatosclerosis

## Abstract

Introduction

Chronic venous insufficiency (CVI) is characterized by inadequate functioning of venous valves in the lower limb. CVI is associated with a significant reduction in patient’s quality of life (QOL). The severity of CVI was determined by CEAP (clinical, etiological, anatomical, pathophysiological) classification and venous clinical severity score (VCSS). The study is aimed to evaluate and correlate Dermatology Life Quality Index (DLQI) with VCSS, CEAP in patients with CVI.

Methods

A cross-sectional study of 57 patients with CVI was conducted over a period of 12 months. A sociographic survey, clinical and severity grading using CEAP classification, and VCSS were done for all venous doppler confirmed patients. QOL was evaluated by validated DLQI questionnaires using English and native languages Hindi and Marathi.

Results

A total of 57 patients with a male to female ratio of 6.1:1 and a mean age of 51.68 years were included in the study. CEAP grading in patients showed 49.12% (C4a), 21.05% (C6), 15.7% (C4b), 7.01% (C3), 3.50% (C2 and C5). Mean VCSS and DLQI were 11.47 and 10.12, respectively; 49.12%, 40.35%, 10.53% of patients had a moderate, very large, and small impact on DLQI respectively, positively correlating to VCSS (P < 0.001).

Conclusion

From this study, it was observed that VCSS and CEAP positively correlated with DLQI, and the impact increases in proportion with the seriousness of the disease.

## Introduction

Chronic venous disease is defined as abnormalities of the venous system’s morphology and functions, which are presented by various clinical signs and manifestations suggesting the necessity for investigation and management. The word chronic venous insufficiency (CVI) is used for leading signs of venous hypertension that have advanced to edema, various alterations in the skin, and venous ulcers [1]. Many factors like venous stasis, venous hypertension, fibrin cuff, and leukocyte trapping influence chronic venous insufficiency. The incidence of CVI varies from less than 1-40% in women and 1-17% in men whereas for varicose veins it is 1-73% in women and 2-56% in men [2]. Predisposing factors comprise old age, more than two pregnancies, genealogy of venous disease, obesity, female sex, sedentary lifestyle, long-standing at work causing orthostasis [2-3]. CVI in the leg causes multiple dermatological manifestations like varicose veins, venous ulcers, lipodermatosclerosis, induration, edema, venous eczema [4-6]. To standardize these manifestations of CVI, clinical, etiological, anatomical, and pathophysiological (CEAP) classification was developed by the American Venous Forum in 1994 [7-9]. CEAP lacks the ability to assess the disease severity hence a venous clinical severity scoring system (VCSS) was established by the American Venous Forum in March 2000 [9].

VCSS is a dynamic scoring system consisting of 10 components which are pain, varicose veins, edema, pigmentation, inflammation, induration, ulcer size, number of ulcers, duration of ulcer, and use of stockings which are graded from zero, one, two, three corresponding to absent, mild, moderate and severe respectively to produce a 30-point maximum score [10-11]. Finlay and Khan developed Dermatology Life Quality Index (DLQI) in the year 1994 and it comprises a 10-item questionnaire that covers seven domains: symptoms, feelings, daily activities, leisure items, work and school, personal relationship, treatment. Skin disorders have been acknowledged to have a detrimental effect on the quality of life of patients. This psychosocial aspect of skin disease has major implications which need to be addressed for optimal management of patients. The DLQI is estimated by totaling up the outcomes of each question, giving rise to a maximum score of 30 and a minimum score of 0, with the score being directly correlating with the impairment [12]. The study is aimed to evaluate and correlate DLQI with VCSS, CEAP in patients with chronic venous insufficiency.

## Materials and methods

This cross-sectional study was done over a period of one year from November 2019 to October 2020 among 57 patients with chronic venous insufficiency in a tertiary care center. Venous Doppler ultrasound scanning was performed in all patients after institutional ethics committee approval and informed consent from patients, VCSS was evaluated (Table 1) and clinical grading was performed using CEAP classification (Table 2). The data regarding age, sex, height, weight, BMI, occupation, level of daily physical activity, duration of standing was recorded.

**Table 1:** Venous Clinical Severity Score (VCSS)

Attributes	Absent = 0	Mild = 1	Moderate = 2	Severe = 3
Pain	None	Occasional	Daily not limiting	Daily limiting
Varicose veins	None	Few	Multiple	Extensive
Venous edema	None	Foot and ankle	Below knee	Knee and above
Skin pigmentation	None	Limited (old)	Diffuse (recent)	Wider (recent)
Inflammation	None	Mild cellulitis	Moderate cellulitis	Severe cellulitis
Induration	None	Focal <5 cm	<1/3 of a gaiter	>1/3 of a gaiter
Number of active ulcers	None	One	Two	More than two
Active ulcer duration (diameter)	None	<3 months	3-12 months	>1 year
Active ulcer size	None	<2 cm	2-6 cm	.>6 cm
Compressive therapy	None	Intermittent	Most days	Full compliance

**Table 2:** CEAP classification CEAP: Clinical, Etiological, Anatomical, Pathophysiological Classification

C0	No visible or palpable signs of venous disease
C1	Telangiectasias or reticular veins
C2	Varicose veins
C3	Edema
C4a	Pigmentation or eczema
C4b	Lipodermatosclerosis or Atrophie blanche
C5	Healed venous ulcer
C6	Active venous ulcer

The patients were given DLQI questionnaires comprising 10 open-ended questions in English and the native languages of Hindi and Marathi. Patients were asked to complete the questionnaires based on their experiences from the past week. DLQI has four different responses: ‘not relevant, ‘not at all’, ‘a little’, ‘a lot’ or ‘very much’ which are graded from zero to four which then is evaluated as 0-1, 2-5, 6-10, 11-20, 21-30 which correlates to no effect, small effect, moderate effect, very large effect, extremely large effect, respectively (Table 3).

**Table 3:** Interpretation of DLQI score DLQI: Dermatology Life Quality Index

0-1	No effect at all on the patient’s life
2-5	Small effect on patient’s life
6-10	Moderate effect on patient’s life
11-20	Very large effect on patient’s life
21-30	Extremely large effect on patient’s life

All the collected data were analyzed by using SPSS V.18 (SPSS Inc., Chicago, IL). Correlation between VCSS and DLQI was assessed by Spearman’s rank correlation coefficient. To investigate the relationship between VCSS and impact on quality of life (QoL), logistic regression was performed. P-value <0.05 is observed ‘to indicate a statistically significant difference.

Inclusion criteria: Clinically and Venous Doppler confirmed cases of CVI who have not received treatment in the last six months.

Exclusion criteria: Age <18 years, arterial disease, deep vein thrombosis, congenital venous disease.

## Results

A total of 57 patients were included in this study. The age of patients ranged from 26 to 80 years with a mean of 51.68 ± 14.53 years. Most of the patients were between the age of 40-60 years (40.35%). 26.31% and 33.33% of patients were between age 20-40 years and 60-80 years, respectively. The mean duration of the disease was 45.91 ± 9.41 months. Most of the patients were males (86%) with a ratio of male to female being 6.1:1. The average BMI was 27.18 ± 4.33 with 51% being overweight, 19% being obese and 30% being normal weight. Almost all patients gave a history of long-standing hours at work. Half of the female patients (four females) in this study had three or more pregnancies. Occupational diversification among the study group showed predominant involvement of security guards (15.8%), followed by shopkeepers (12.3%) and others (Table 4).

**Table 4:** Occupation wise distribution of patients

Profession	Number	%
Security guard	9	15.78%
Shop keeper	7	12.28%
Miscellaneous	6	10.5%
Hotel worker	5	8.77%
Housewife	5	8.77%
Driver	4	7.01%
Construction worker	4	7.01%
Conductor	3	5.26%

**Figure 1:**
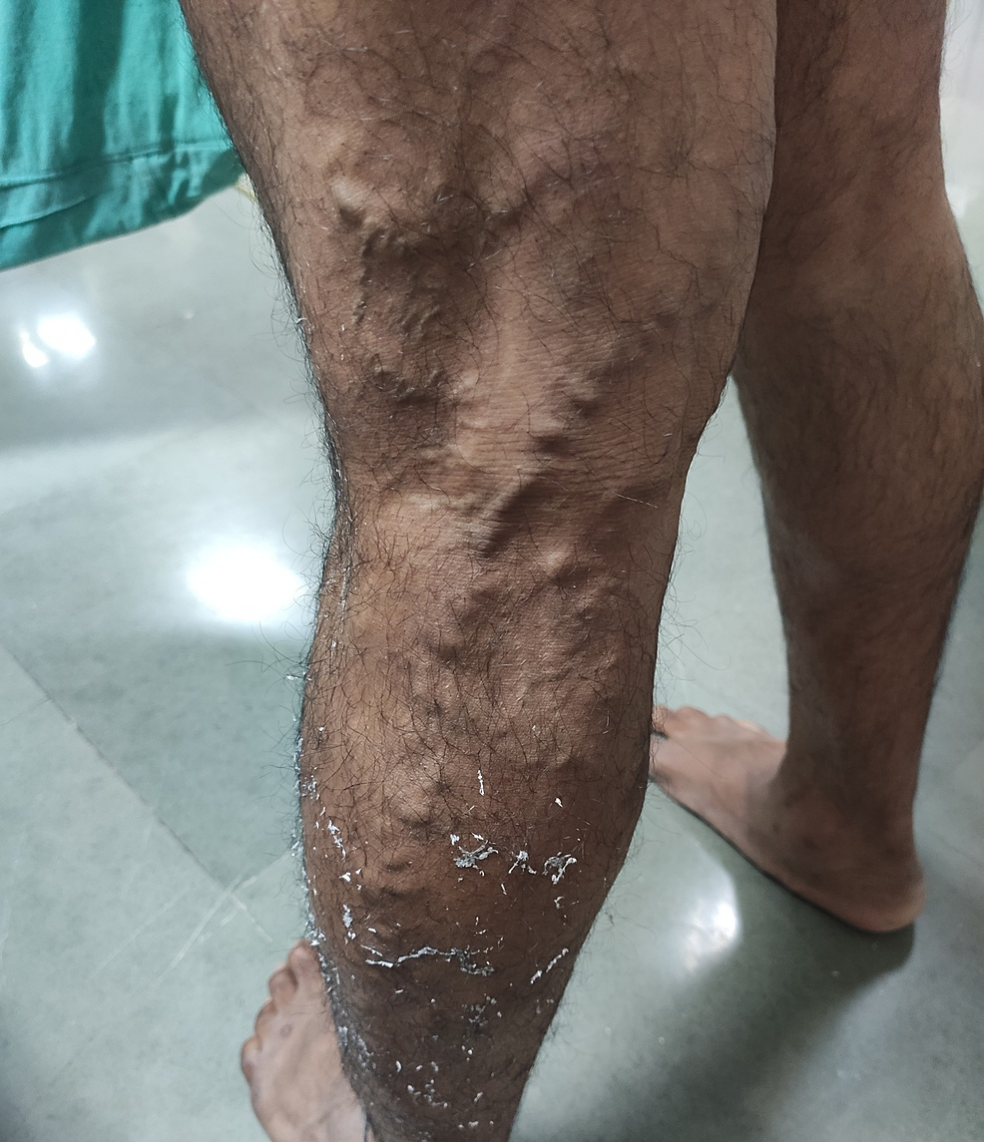
Varicose veins in a 50-year-old male in the left lower limb

**Figure 2:**
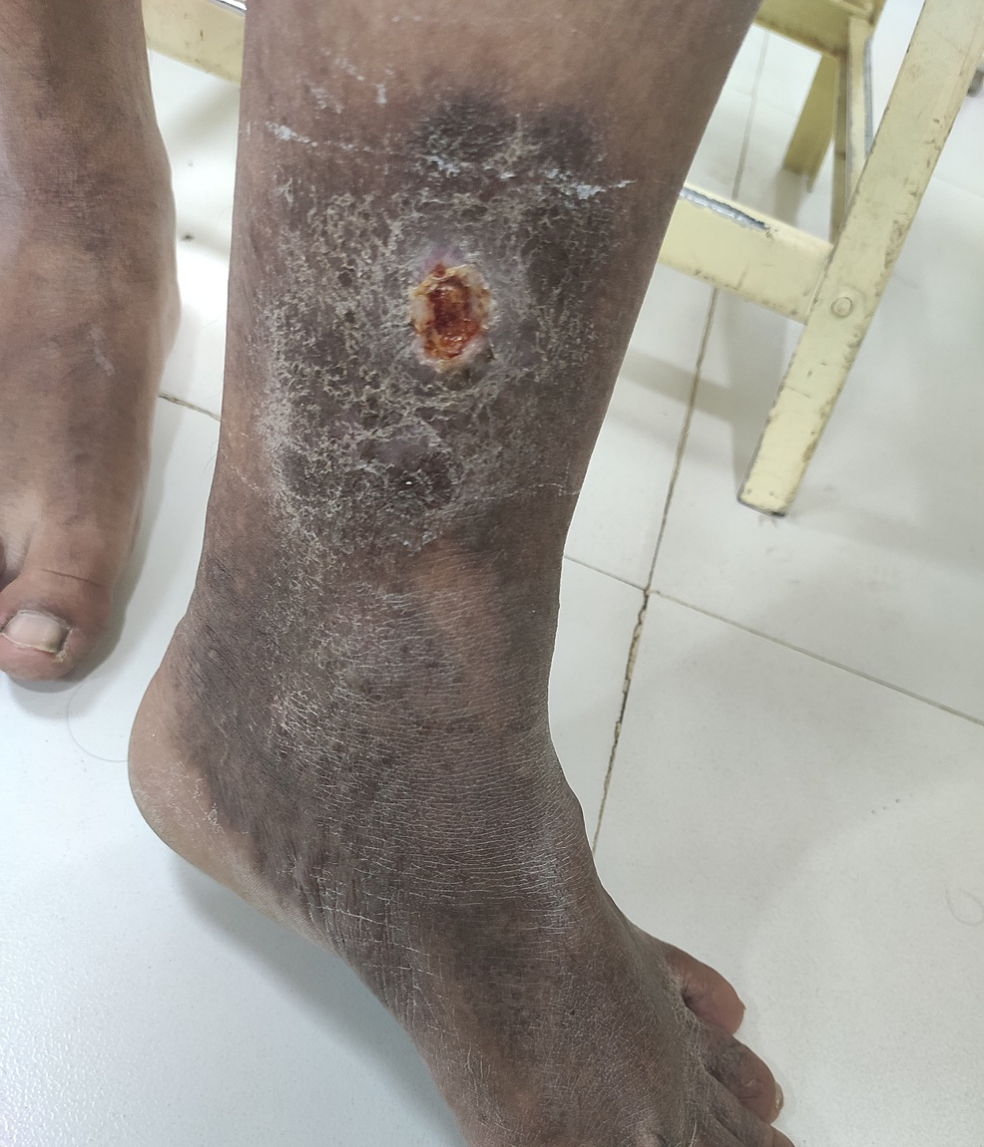
Venous ulcer with surrounding hyperpigmentation above the left medial malleolus in a 65-year-old male

Among 57 patients in our study, there were two patients (3.50%) of CEAP grade C2, four patients (7.01%) of grade C3, 28 patients (49.12%) of grade C4a, nine patients (15.7%) of grade C4b, two patients (3.50%) of grade C5, 12 patients (21.05%) of grade C6. Among the 10 aspects of VCSS, varicose veins (Figure 1) were present in all patients. Moderate pain and varicosity were the most common findings seen in 27 patients (47.37%), followed by moderate venous edema in 24 patients (42.10%). Twenty-six patients (45.61%) had moderate skin pigmentation. Moderate inflammation and moderate use of compression therapy were seen in 22 patients (38.60%) and 16 patients (28.07%), respectively. Moderate induration was seen in 14 patients (24.56%). Active leg ulcerations (Figure 2) were seen in 21.05% of patients. Single, double, and multiple ulcerations were seen in 5.26%, 10.53%, 5.26% of patients, respectively. Ulcers lasting for more than three months were seen in 10 patients (17.54%), of which 8.77% of patients had ulcers persisting for more than a year. Eight patients (14.04%) had ulcers of size more than 2 cm and four patients (7.02%) had ulcers of size less than 2 cm. Compliance with compression therapy was seen in 38 patients (66.67%), of which only one patient (1.75) showed full compliance. Distribution of different attributes of VCSS is given in Table 5.

**Table 5:** Distribution of different attributes of VCSS (n = 57) VCSS: Venous Clinical Severity Score

Attributes	Absent = 0	Mild = 1	Moderate = 2	Severe = 3
Pain	5 (8.77%)	20 (35.09%)	27 (47.37%)	5 (8.77%)
Varicose vein	0	12 (21.05%)	27 (47.37%)	18 (31.58%)
Venous edema	5 (8.77%)	17 (29.82%)	24 (42.10%)	11 (19.30%)
Inflammation	11 (19.30%)	22 (38.60%)	22 (38.60%)	2 (3.51%)
Skin pigmentation	6 (10.53%)	16 (28.07%)	26 (45.61%)	9 (15.79%)
Induration	34 (59.65%)	2 (3.51%)	14 (24.56%)	7 (12.28%)
Number of active ulcers	45 (78.95%)	3 (5.26%)	6 (10.53%)	3 (5.26%)
Duration of ulcer (months)	45 (78.95%)	2 (3.51%)	5 (8.77%)	5 (8.77%)
Size of ulcer (cm)	45 (78.95%)	4 (7.02%)	8 (14.04%)	0
Compression therapy	19 (33.33%)	21 (36.84%)	16 (28.07%)	1 (1.75%)

**Figure 3:**
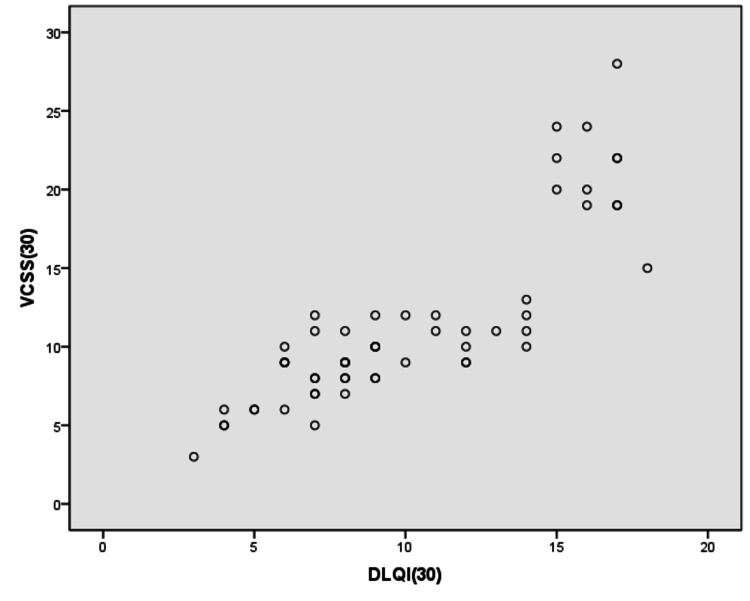
Scatter plot showing the correlation between DLQI and VCSS DLQI: Dermatology Life Quality Index; VCSS: Venous Clinical Severity Score.

VCSS of the patients in this study ranged from 3-28 with a mean of 11.47 ± 5.63. DLQI of the patients ranged from 3-18 with a mean of 10.12 ± 4.16. Six patients (10.53%), 28 patients (49.12%), 23 patients (40.35%) had a small, moderate, and very large effect on their daily lives, respectively. None of the patients had any effect or an extremely large effect on DLQI. A strong positive correlation was found between DLQI and VCSS in which the value of Spearman’s rank correlation coefficient 'r' = 0.840 and the ‘p' value obtained is <0.05 (Figure 3).

## Discussion

Chronic venous insufficiency is often accompanied by distinguishing (varied)/various dermatological outcomes/features. Identification of these cutaneal findings is extremely advantageous in detecting the most frequently seen chronic vascular diseases and starting early management [1]. The CEAP classification was developed by an international consensus conference for the notification of diagnostic details in chronic venous disease, as well as for routine patient documentation and treatment [3, 13-14]. DLQI is the first dermatology-specific quality of life questionnaire published to measure dermatological distress [15-19]. In the Edinburg vein study, the prevalence of CVI is 9.4% in men and 6.6% in women [20]. In this study, the proportion of males was higher than females (6.1:1). A study done by Mallick et al. [15] in 2020 showed VCSS positively correlating with DLQI and CVI exerting small to moderate effect on patient’s QoL, whereas in our study it showed moderate to very large effect. In a study done by Gillet et al. [21] on 60 patients, it was found that increased VCSS values directly correlate with increased CEAP scores. Ricci et al. evaluated the VCSS in 210 patients (420 limbs) where VCSS was 0 in 283 limbs, and VCSS was 1 or greater in the following categories: pain, varicose veins, edema, skin pigmentation, inflammation, induration, and compressive therapy in 63, 70, 51, 17, 2, 8, 9 limbs, respectively. This study was done to estimate the gravity of the disease and to substantiate the VCSS results with the ultrasonographic findings and observed the potentiality of the VCSS to act as a screening tool for CVI which could recognize patients at a higher risk for complications and with severe disease [22].

Epidemiologic studies have shown that varicose veins are more common in patients with increased body mass index (BMI) (especially >30 kg/m^2^) [3], as it was also seen in our study that 51% of patients being overweight and 19% being obese but 30% of patients in our study also had normal BMI indicating the role of multiple predisposing factors in the pathogenesis of CVI. In the study done by Lullove and Alvarez [23], varicosity, skin pigmentation, induration, and inflammation were the frequently noticed findings but in our study pain, varicose vein, venous edema were the most common findings. In our study, the presence of active venous ulcer was found in 21.05% of cases; amongst them, ulcers with the largest dimension of 2-6 cm (14.04%) and nonhealing ulcers persisting for 3-12 and >12 months were found in maximum patients. But the study done by Vasquez et al. found active ulcers only in 8% of cases, and small ulcers (<2 cm) and large ulcers (2-6 cm) were equally common, and nonhealing ulcers for 3-12 months were frequently observed findings. In the study done by Vasquez et al., compliance to compression therapy was higher (98.9%) as compared to our study (66.67%) [24]. This study was done to document various dermatological manifestations of chronic venous insufficiency and grade their severity with CEAP score and correlate Venous Clinical Severity Score (VCSS) with Dermatology Life Quality Index (DLQI) to prevent further complication and early management and to understand the psychosocial impact on patients. Kahn et al. [25] showed in their study that the higher the CEAP class lower was the SF-36 Physical Component Summary scores and VEINES-QOL with no effect in SF-36 mental Component Summary. In our study, it was found that VCSS showed a strong positive correlation to DLQI, and hence it can be elucidated that the higher the VCSS, the higher is the effect on the quality of life. DLQI, therefore, helps in assessing the effectiveness of treatment in the patients and their emotional aspect to the disease addressing the need to give counseling to patients regarding the same. Our study shows a positive correlation and a linear relationship between DLQI and VCSS, thereby helping the overall patient satisfaction once the issues are addressed and properly taken care of.

The main limitation in using the CEAP classification is that it responds poorly to changes over time and many of its components are relatively static and do not change significantly after treatment [25]. The advantage of using VCSS over CEAP is the avoidance of static elements and the use of only those that had the ability to reflect changes over time. It is hence recommended to use both the scoring system while evaluation rather than using one or the other [10]. In our study, we have used both the scoring systems and correlated with the quality of life. Few limitations in this study are the small sample size, which represents data from a specific region. DLQI was only translated to three languages in this study, hence limiting the patients from other linguistic states. The inclusion of control groups and a large sample size would have added more value to the study and its outcomes.

## Conclusions

Chronic venous insufficiency adversely affects the quality of life by influencing the psychosocial wellbeing of the patients. In our study, mean VCSS positively correlates with DLQI in patients with CVI. Hence more severe the disease, the more it affects the DLQI. Assessment of DLQI in patients with CVI improves the effectiveness of the treatment and also addresses other psychosocial consequences of the disease which need to be managed giving more accurate insight to the clinicians.
